# The Safety of Intralesional Steroid Injections in Young Children and Their Effectiveness in Anastomotic Esophageal Strictures—A Meta-Analysis and Systematic Review

**DOI:** 10.3389/fped.2021.825030

**Published:** 2022-01-28

**Authors:** Annefleur R. L. van Hal, Rebecca Pulvirenti, Floris P. J. den Hartog, John Vlot

**Affiliations:** ^1^Department of Pediatric Surgery and Intensive Care, Erasmus Medical Centre Sophia Children's Hospital, Rotterdam, Netherlands; ^2^Pediatric Surgery Unit, Department of Women's and Children's Health, University Hospital of Padua, Padua, Italy; ^3^Department of Surgery, Erasmus Medical Centre, Rotterdam, Netherlands

**Keywords:** steroids, intralesional steroid injections, esophageal strictures, esophageal atresia (EA), esophageal dilatation, safety, effectiveness

## Abstract

**Objective:**

Intralesional steroid injections (ISI) are a widely used technique for various pediatric indications and represent a possible adjuvant treatment for anastomotic esophageal strictures. Yet, no consensus has been reached neither on their safety in the pediatric population or their effectiveness in esophageal atresia patients. This systematic review aimed to assess the safety of ISI in young children through a meta-analysis and to summarize the current knowledge on the effectiveness of ISI in anastomotic esophageal strictures.

**Methods:**

A systematic literature search was performed in Embase, Medline, Web of Science Core Collection, Cochrane Central Register of Controlled Trials and Google Scholar up to August 16 2021. Studies focusing on ISI and involving children up to 2 years were included in the meta-analysis for the safety assessment. All studies evaluating the use of ISI as adjuvant treatment in anastomotic esophageal strictures in children were included in the systematic review to assess the effectiveness of the intervention.

**Results:**

The literature search yielded 8,253 articles. A total of 57 studies were included, of which 55 for the safety and five for the effectiveness assessment. The overall complication rate was 7%, with a greater incidence of local complications compared to systemic complications. Six studies (with a total of 367 patients) evaluated adrenocorticotropic hormone and cortisol levels, of which four reported hypothalamic-pituitary axis suppression. Two children (0.6%) received replacement therapy and all patients recovered uneventfully. A mean number of 1.67 ISI were performed per esophageal atresia (EA) patient. A reduction of needed dilatations was seen after ISI, compared to the number of dilatations performed before the intervention (5.2 vs. 1.3).

**Conclusion:**

The insufficient data emphasized the need for further prospective and comparative studies. Results from this meta-analysis and systematic review address ISI as a safe and effective technique. Close clinical follow-up and growth curve evaluation are advisable in patients receiving ISI.

**Systematic Review Registration:**

PROSPERO, identifier: CRD42021281584.

## Introduction

The prognosis of esophageal atresia (EA) patients has greatly improved over the past decades, due to advances in surgical techniques as well as pre- and postoperative care (1, 2). With the higher survival rate, postoperative morbidity has increasingly become an issue, creating a substantial burden for patients and their families. One of the main short- and long-term complications after EA repair are anastomotic strictures, with an incidence of up to 60% (3). These strictures following EA repair also represent the most common cause of esophageal strictures in the pediatric population (4), exceeding the chemical burns (5).

Several therapeutic options are available for the management of esophageal strictures, yet none have been proven highly effective or risk-free. Non-surgical treatment is commonly preferred over surgical treatment and esophageal dilatations are considered the gold standard (6, 7). In the last decades several endoscopic procedures have been developed as adjuncts to dilatations (4, 8), such as endoscopic incisional therapy (9), placement of externally removable stents (10), mitomycin C injections (11), and intralesional steroid injections (ISI) (12).

Such treatments are usually reserved for recurrent or refractory strictures which respond poorly to dilatations. The ESPGHAN (European Society for Pediatric Gastroenterology Hepathology and Nutrition) refers to recurrent strictures when three or more clinically relevant stricture relapses occur despite dilatations; conversely, a refractory esophageal stricture is defined as an anatomic restriction without endoscopic inflammation that results in dysphagia after a minimum of five dilatation procedures at maximally 4-week intervals (13, 14).

Intralesional steroid injections are gaining popularity for different medical indications, both in adults and in pediatric patients, and tend to show promising results (16, 17). The rationale behind this technique is the capability of steroids to locally inhibit the inflammatory response and to reduce the collagen and fibroblasts deposit during the scar healing process. Less inflammation should result in less scarring and tissue contraction, which in the case of esophageal anastomosis translates into a lower rate of stricture development (18–22). Local side effects of ISI, e.g., perforations, intramural, and candida infections, may possibly negatively influence the scar healing process (8).

The main indications for the use of ISI in children and adolescents are keloids, hemangiomas, and juvenile idiopathic arthritis (17, 23, 24). The application of this technique in the treatment of esophageal strictures was firstly described by Ashcraft et al. (25). Since then, not much scientific progress has been made and the technique is still scarcely used, partly due to the lack of standardized protocols. Additionally, prescription of steroids for the use in esophageal strictures in children is still regarded as “off-label,” raising concerns on the safety.

No recommendations or guidelines for any indication regarding the administration of ISI in children exists, hence the safety is not guaranteed. Moreover, available data on the effectiveness of ISI as adjunct treatment for esophageal strictures are not uniform. The lack of evidence, prompted us to perform a systematic review and meta-analysis on the general use of ISI in the pediatric population. Therefore, the aim of the present meta-analysis and systematic review was to, respectively, assess the safety of ISI for any indication in the pediatric population up to 2 years of age and more specifically assess the effectiveness of ISI in patients with esophageal stricture after esophageal atresia repair.

## Methods

### Information Sources and Search Strategy

This review was performed according to an a priori designed protocol and recommended for systematic reviews and meta-analysis (26, 27). Additionally, the principles of the “preferred reporting items for systematic reviews” (PRISMA) statement were adhered to (28). This study is registered in the PROSPERO database (registration number CRD42021281584).

A systematic literature search was performed in Embase, Medline, Web of Science Core Collection, Cochrane Central Register of Controlled Trials, and Google Scholar until August 16 2021. The search strategy is attached in the Supplementary Material. The search and selection criteria were restricted to English language articles and limited to humans. No publication year restriction was considered. Reference lists of relevant articles and reviews were manually searched for additional reports. The results for the meta-analysis and systematic review were retrieved from the same search strategy.

### Inclusion and Exclusion Criteria

Studies were assessed according to the following criteria: population characteristics, intervention, and reported outcome. Different inclusion criteria were used for each primary aim. In the meta-analysis on the safety of ISI, all studies evaluating ISI in children aged up to 2 years were included. The age cut-off was set to obtain a study population as representative as possible of the average patient receiving ISI as adjuvant treatment for esophageal stenosis. Studies reporting an aggregate age of the patients were included when the mean age was under 2 years and the age range between 0 and 5 years.

In the systematic literature search concerning the effectiveness of ISI in esophageal strictures, studies evaluating ISI in all children with a history of EA repair were included. Studies comparing ISI and other interventions were included, as were those administering ISI with co-medication (e.g., propranolol, bleomycin). Studies focusing on different types of steroid administration (IV, oral, topical) were manually excluded. Conference abstracts, editorials, letters, short surveys, studies reporting non-original data (systematic reviews, meta-analysis, narrative reviews) and unavailable full-text articles were excluded. Absence of discrete patient-data was an additional exclusion criterion.

### Study Selection

Two review authors (AH and RP) independently screened titles and abstracts to select eligible studies. Disagreements about study selection were resolved by discussion. AH and RP screened full-texts of the selected studies against the inclusion and exclusion criteria. During all stages of study selection, any uncertainties or discrepancies were discussed until consensus was achieved. If consensus was not reached, disagreements were resolved by discussing them with a senior researcher (JV).

### Methodological Quality

The included studies were reviewed independently by AH and RP on methodological quality according to the Joanna Briggs institute (JBI) critical appraisal tool for case reports, case series, cohort studies, and quasi-experimental studies; depending on the type of study (29–31). The JBI form contains, respectively, 8, 10, 11, and 9 items, each of which is assessed as “yes,” “no,” “unclear,” or “not applicable.” Items scored as a “yes” are assigned a score 1; other assessments are not assigned a score. The obtained scores could not be linked to a standardized judgement tool. For case reports the total range varied between 0 and 8. Within the research team it was concluded that six to eight points were regarded as high quality; 4–5 points moderate quality; and 1–3 points low quality. For case series, with total range between 0–10, 8–10 was defined as high quality; 5–7 moderate quality, and 1–6 low quality. Subsequently, quasi-experimental studies, with total range between 0–9, were divided into the following groups: 7–9 high quality, 4–6 moderate quality, and 1–3 low quality. Finally, for cohort studies with a total range between 0–11 was concluded that 9–11 would be high quality; 5–8 moderate quality and 1–4 low quality. The critical appraisals by AH and RP were crosschecked; any differences were discussed until consensus was achieved. If consensus was not reached, disagreements were resolved by discussing these issues with JV.

### Data Extraction

The following variables were extracted and entered into a standard data extraction form: author, publication year, country treating hospital, study type, number of included patients, patient's age at time of the injections, intervention description, type of steroid used, dosage, number of injections, time interval between injections, co-medication, length of follow-up, local side effects, systemic side effects, need for treatment of side effects, and whether the adrenocorticotropic hormone (ACTH) or cortisol levels had been measured.

Moreover, regarding the evaluation of the effectiveness, the following information was gathered: type of EA according to Gross (32), stricture length, symptoms, mean number of dilatations prior to and post injection, type of dilatation technique used, need and time for further dilatation, side effects, and possible complications.

### Synthesis

Triamcinolone is the most commonly used steroid in the treatment of esophageal strictures. Hence, all steroid dosages were converted to triamcinolone-dose equivalents for the comparison of the administered steroid dosages. The safety of corticosteroid injections was assessed by splitting the patients in groups receiving a higher (>80 mg) and lower (≤80 mg) first dosage of triamcinolone acetonide (TA). This decision is based on the Dutch (College ter Beoordeling van Geneesmiddelen−06/09/2021) and Italian (Agenzia Italiana del Farmaco−17/01/2018) summary of product characteristics. Both documents state that single injections at multiple sites up to a total amount of 80 mg have been administered without severe side effects. When multiple injections were given, the dosage of the first injection was used to group the patients. In addition, when assessing the dosage, some of the included studies did not show the total amount of received corticosteroid, yet only the amount of milligrams per kilogram body weight administered. In all cases, it could be assumed that the patients did not exceed the dosage of 80 mg as children <2 years will not weigh more than 40 kg.

Statistical analysis was performed with SPSS 25.0 (IBM, Armonk, NY, USA) and R (version 4.01) (33). Aggregated continuous baseline variables were calculated as means or medians of extracted variables from the included studies. Categorical and continuous variables were summarized as numbers with percentages. Using a generalized linear mixed model, pooled proportions of outcomes were calculated. The 95% confidence interval (95% CI) was calculated. Heterogeneity was quantified using the τ^2^-characteristic, which was calculated using the maximum likelihood method. The Knapp-Hartung adjustment was applied. A prediction interval was given, which indicates the interval in which a single future observation will fall, given what has been observed. In order to perform such analysis, studies not stating the mean number of injections or the steroid dosage, were not included. Additionally, all studies involving <three patients were excluded from this meta-analysis. In order to assess potential publication bias, a funnel plot was created. A two sided *p* < 0.05 was considered statistically significant.

A subgroup of studies mentioning systemic complications was evaluated for age, dosage, number of injections, co-medication, and the need for treatment. The same outcome parameters were evaluated for subgroups involving studies referring to ISI-related endocrine complications and studies measuring cortisol and ACTH blood levels.

## Results

**Figure 1:**
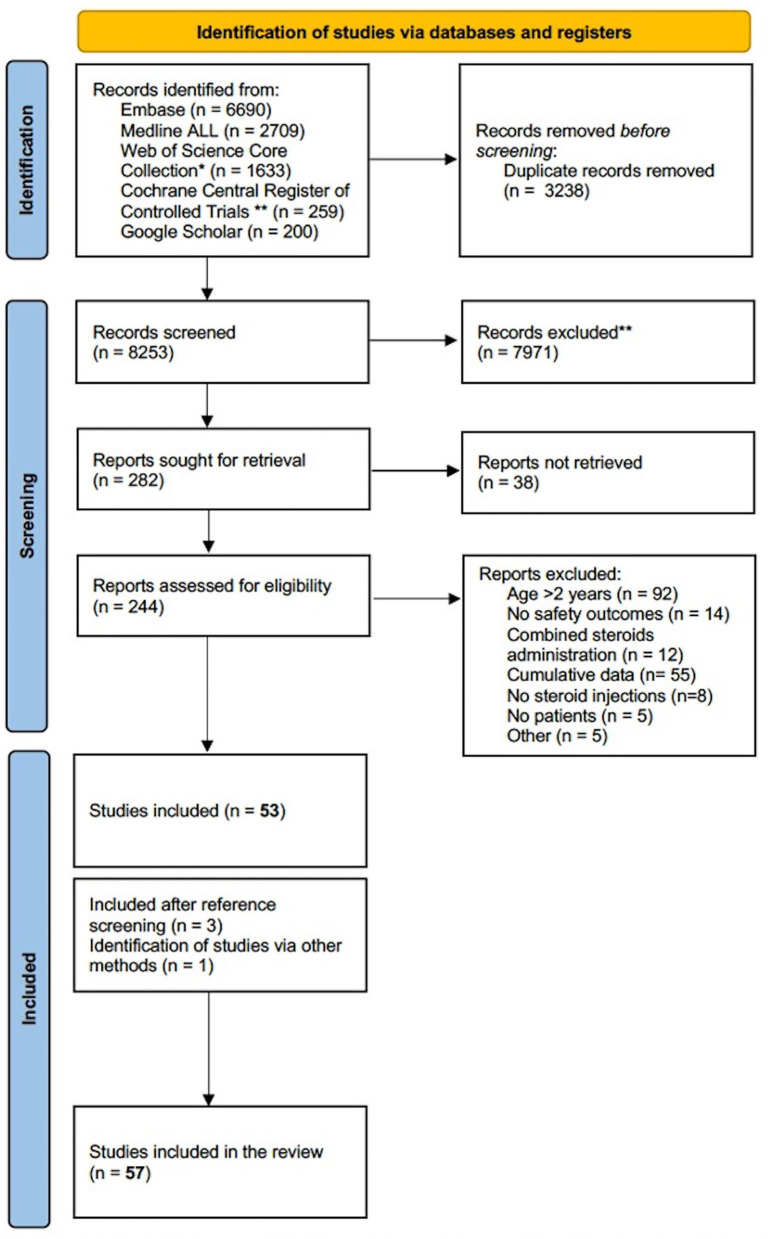
PRISMA flow diagram of literature search (15). *Science Citation Index Expanded (1975-present); Social Science Citation Index (1975-present); Arts and Humanities Citation Index (1975-present); Conference Proceedings Citation Index-Science (1990-present); Conference Proceedings Citation Index- Social Science and Humanities(1990-present); Emerging Sources Citation Index (2015-present). **Manually deleted abstracts from trial registries.

The systematic search strategy yielded 8,253 articles, of which 244 were further assessed for eligibility. After full-text screening, 53 articles were selected for inclusion. Reference screening of the included studies yielded three additional studies suitable for inclusion. One study, discussing ISI for anastomotic esophageal strictures, was not retrieved from the search strategy, yet included in the systematic review since it complied with all inclusion criteria (34) (Figure 1).

Fifty-five studies, amounting to 3,499 patients who underwent one or several ISI were included for the general safety assessment (12, 34–87). Five studies were included for the effectiveness assessment of ISI in the treatment of esophageal strictures, comprising 173 esophageal atresia patients (12, 34, 47, 88, 89).

Clinical indications for the ISI varied between patients, with cutaneous hemangioma being the most common with a total of 3,078 patients. Characteristics of the included studies are shown in Table 1.

**Table 1:** Characteristics of included studies.

**References**	**Year**	**Country**	**Study type**	**Number of included patients**	**Treatment indication**	**JBI critical appraisal score**	**JBI quality verdict**
Abe (61)	1986	Japan	Case Report	2	Cutaneous hemangioma	4/8	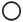 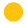 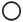
Al-Mahdi (62)	2010	Qatar	Case Report	1	Cutaneous hemangioma	5/8	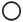 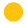 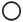
Alsman and Mounir (35)	2017	Egypt	Quasi-experimental	33	Periocular hemangioma	7/9	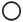 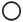 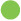
Bonavolontà et al. (36)	1985	Italy	Case series	6	Cutaneous hemangioma	8/10	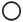 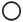 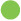
Bonavolontà^Δ^ et al. (36)				9			
Buckmiller et al. (37)	2008	USA	Case series	21	Parotid hemangioma	7/10	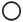 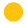 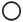
Couto and Greene (42)	2014	USA	Case series	100	Cutaneous hemangioma	6/10	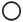 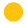 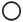
Chai et al. (38)	2019	China	Case series	1039	Cutaneous hemangioma	9/10	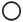 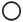 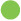
Chantharatanapiboon (39)	2008	Thailand	Case series	139	Cutaneous hemangioma	8/10	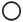 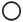 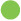
Chen et al. (40)	2000	Taiwan	Case series	155	Cutaneous hemangioma	7/10	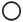 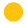 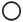
Colberg et al. (41)	2008	Puerto Rico	Case series	6	Synovial cyst	6/10	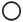 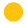 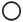
Droste et al. (63)	1988	USA	Case report	2	Periocular hemangioma	4/8	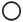 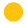 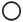
Edmonson and Bent (64)	2010	USA	Case report	1	Subglottic stenosis	7/8	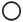 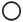 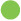
Emir et al. (43)	2015	Turkey	Case series	6	Cutaneous hemangioma	5/10	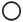 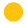 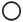
Folia et al. (65)	2007	France	Case report	1	Glottic hemangioma	2/8	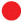 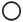 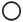
Fonseca et al. (66)	2021	Chile	Case report	1	Nodular fasciitis	4/8	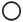 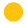 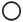
Gandhi et al. (88)	1989	USA	Quasi-experimental	5	Esophageal stricture	4/8	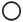 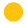 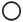
Gangopadhyay et al. (44)	1996	India	Case series	105	Periocular hemangiomas	4/10	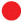 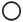 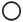
Gorst et al. (67)	2001	UK	Case report	1	Periorbital hemangioma	2/8	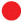 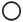 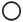
Goyal et al. (45)	2004	UK	Case series	4	Periocular hemangioma	8/10	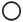 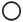 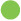
Helal and Daboos (46)	2019	Egypt	Quasi-experimental	340	Cutaneous hemangioma	7/9	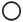 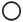 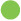
Hoeve et al. (68)	1997	The Netherlands	Case series	11	Subglottic hemangioma	5/8	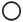 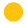 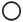
Holder et al. (47)	1969	USA	Quasi-experimental	4	Esophageal stricture	2/9	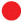 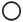 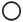
Hoornweg et al. (69)	2014	The Netherlands	Cohort study	29	Periorbital hemangioma	10/11	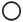 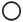 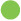
Janmohamed et al. (70)	2011	The Netherlands	Case series	34	Periocular hemangioma	9/10	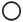 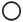 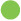
Kang and Kim (71)	2002	Korea	Case report	1	Mastocitoma	3/8	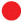 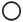 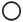
Khamalrudin and Goh (72)	2021	Malaysia	Case report	1	Glottic hemangioma	6/8	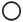 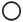 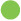
Kushner (48)	1982	USA	Case series	9	Periocular hemangioma	8/10	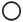 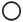 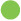
Kushner (49)	1985	USA	Case series	21	Periorbital hemangioma	7/10	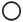 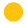 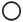
Kushner^Δ^ (49)				3			
Langmann and Lindner (50)	1994	Austria	Case series	4	Periocular hemangioma	7/10	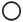 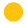 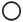
Mazzola (51)	1977	Italy	Case series	11	Cutaneous hemangioma	5/10	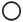 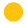 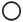
Meeuwis et al. (52)	1990	The Netherlands	Case series	6	Subglottic hemangioma	7/10	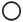 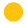 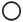
Mohamed (73)	2020	Egypt	Quasi-experimental	26	Cutaneous hemangioma	8/9	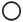 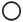 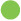
Morkane et al. (53)	2011	UK	Case series	15	Periocular hemangioma	6/10	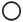 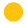 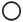
Nelson et al. (74)	1984	USA	Case report	2	Periocular hemangioma	4/8	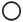 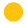 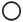
Neumann et al. (75)	1997	USA	Case report	2	Retrobulbar hemangioma	5/8	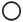 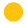 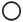
Neumann^Δ^ et al. (75)				1			
Ngo et al. (34)	2020	USA	Case series	158	Esophageal stricture	9/10	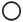 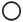 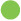
Noe (76)	1981	USA	Case report	2	Urethral stricture	4/8	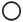 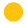 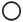
O'Keefe et al. (54)	2003	Ireland	Case series	14	Periocular Hemangioma	7/10	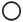 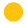 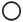
Pandey et al. (55)	2009	India	Quasi-experimental	886	Cutaneous hemangioma	9/9	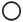 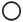 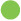
Ragab et al. (56)	2020	Egypt	Case series	25	Cutaneous hemangioma	5/10	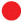 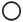 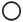
Reyes et al. (77)	1989	Puerto Rico	Case Report	1	Cutaneous hemangioma	5/8	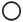 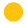 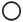
Sabry et al. (78)	2020	Egypt	Quasi-experimental	15	Cutaneous hemangioma	9/9	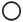 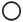 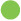
Say et al. (79)	2011	USA	Case Report	1	Periocular hemangioma	4/8	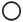 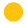 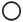
Sekioka et al. (80)	2018	Japan	Case series	1	Subglottic stenosis	10/10	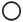 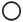 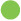
Shao et al. (81)	2016	China	Quasi-experimental	31	Cutaneous hemangioma	8/10	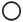 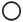 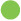
Simic et al. (57)	2009	Serbia	Case series	5	Nasal hemangioma	6/10	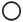 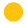 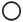
Sun et al. (82)	2020	China	Case series	35	Periorbital hemangioma	4/10	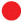 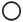 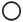
Tasca and Williams (83)	2004	UK	Case report	1	Nasal hemangioma	5/8	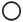 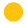 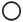
Ten Kate et al. (12)	2020	The Netherlands	Case series	4	Esophageal stricture	10/10	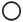 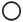 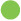
Weiss (84)	1989	USA	Case Report	2	Periocular hemangioma	4/8	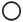 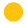 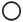
Weiss and Kelly (58)	2008	USA	Case series	13	Periocular hemangioma	8/10	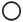 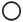 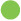
Wilshaw and Deady (59)	1987	UK	Quasi-experimental	15	Vascular hamartomas	3/10	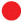 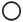 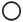
Xu et al. (85)	2018	China	Quasi-experimental	39	Cutaneous hemangioma	7/9	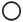 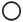 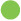
Yuan et al. (60)	2014	China	Case series	16	Periorbital hemangioma	3/10	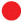 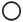 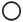
Yuan et al. (86)	2015	China	Quasi-experimental	57	Periocular/nasal ala/ auricular canal hemangioma	4/9	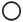 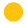 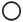
Zein et al. (89)	1995	USA	Quasi-experimental	1	Esophageal stricture	3/9	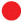 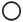 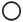
Zhang et al. (87)	2021	China	Case series	36	Cutaneous hemangioma	8/10	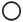 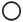 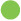

*JBI, Joanna Briggs Institute; USA, United States of America; UK, United Kingdom; Quality verdict: 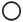 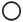 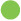, high quality; 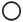 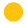 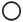, moderate quality; 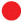 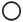 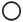, low quality; Δ, Patients receiving ≥80 mg Triamcinolone Acetonide*.

### Study Types and Quality Assessment

A total of sixteen case reports, 28 case series (of which seventeen retrospective), twelve quasi-experimental studies and one cohort study were selected for final inclusion. The quality assessment for case reports provided two high quality, eleven moderate quality and three low quality studies. Eleven high quality, thirteen moderate quality and four low quality case series were assessed. With regard to quasi-experimental studies, seven studies were of high quality, two of moderate quality and three of low quality. The included cohort study was classified as high quality. A summary of the quality assessments for the included studies and their reported outcomes can be found in the Supplementary Material.

**Figure 2:**
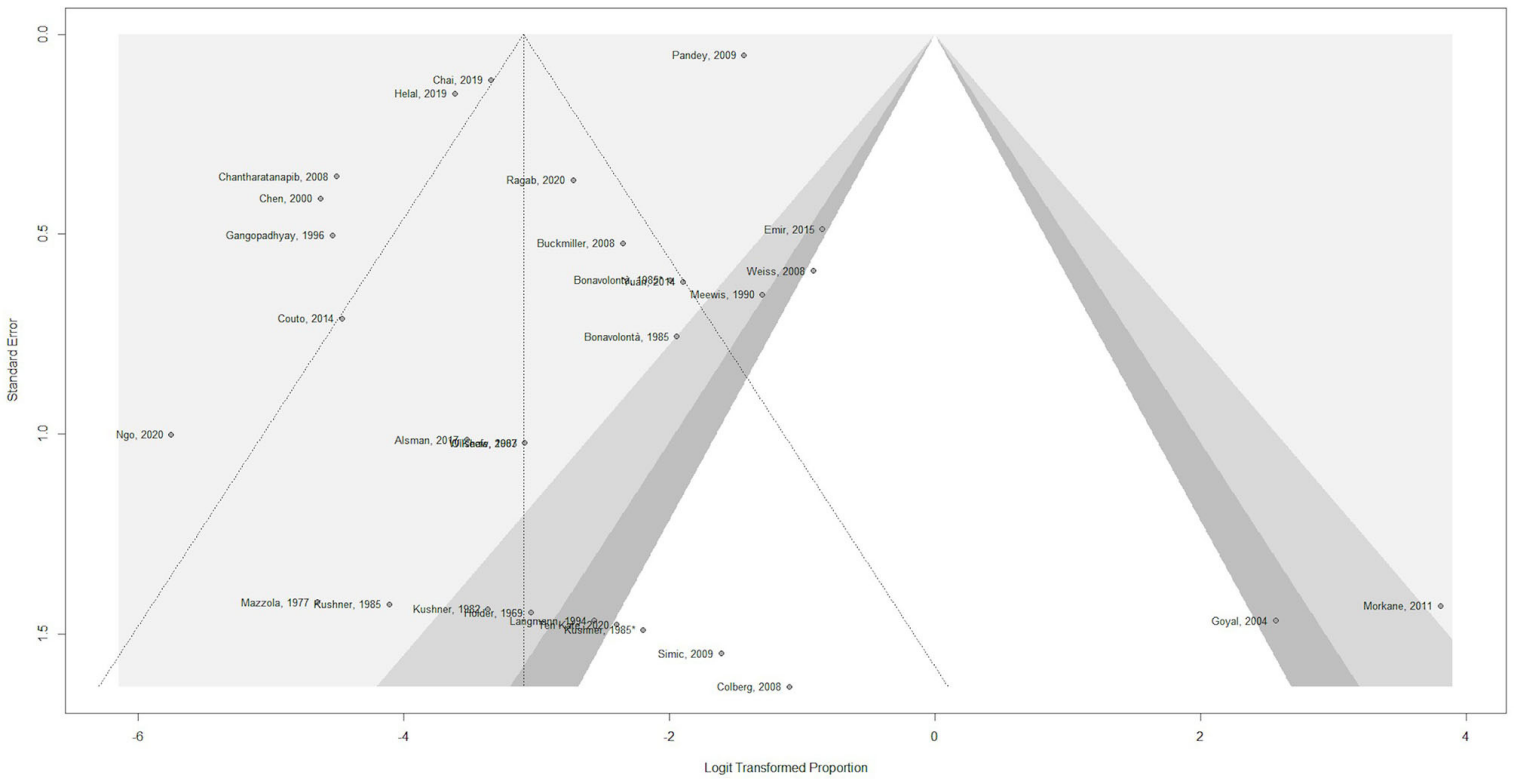
Funnel plot for assessment of potential publication bias (12, 34–60).

A funnel plot was used in order to assess potential publication bias (Figure 2). The x-axis displays the observed incidence of any complication for each of the included studies, normalized by log-transformation in order to be comparable across studies. The y-axis displays the standard error. The outcome considered was the occurrence of any complication, regardless of dosage. There is clear asymmetry, which could indicate potential publication bias, as studies mainly seem to be missing areas of low statistical significance.

### Safety of ISI

#### Patients Characteristics and Therapeutic Approach

The aggregated mean age at time of first ISI, based on data from 52 studies, was 6.91 months (12, 34–38, 40–68, 71–87). Forty-nine studies provided information on the mean number of injections, resulting in 2.9 injections per patient (12, 34–67, 70–72, 74–77, 79, 80, 83–87). The type of steroid injected, mean number of injections and time interval between injections greatly varied among the included studies. Details can be found in the Supplementary Material.

Adverse events following ISI were evaluated. The overall rate for side effects, based on 48 studies and 3,352 patients, was 7.1% (12, 34–67, 70–72, 74–77, 79, 80, 83–87). Six studies mentioned the need for additional treatment (local or systemic) for adverse events (38, 40, 45, 52, 54, 69). The aggregated mean and median follow-up lengths were 17.09 and 12 months, respectively.

#### Systemic vs. Local Side Effects

**Figure 3:**
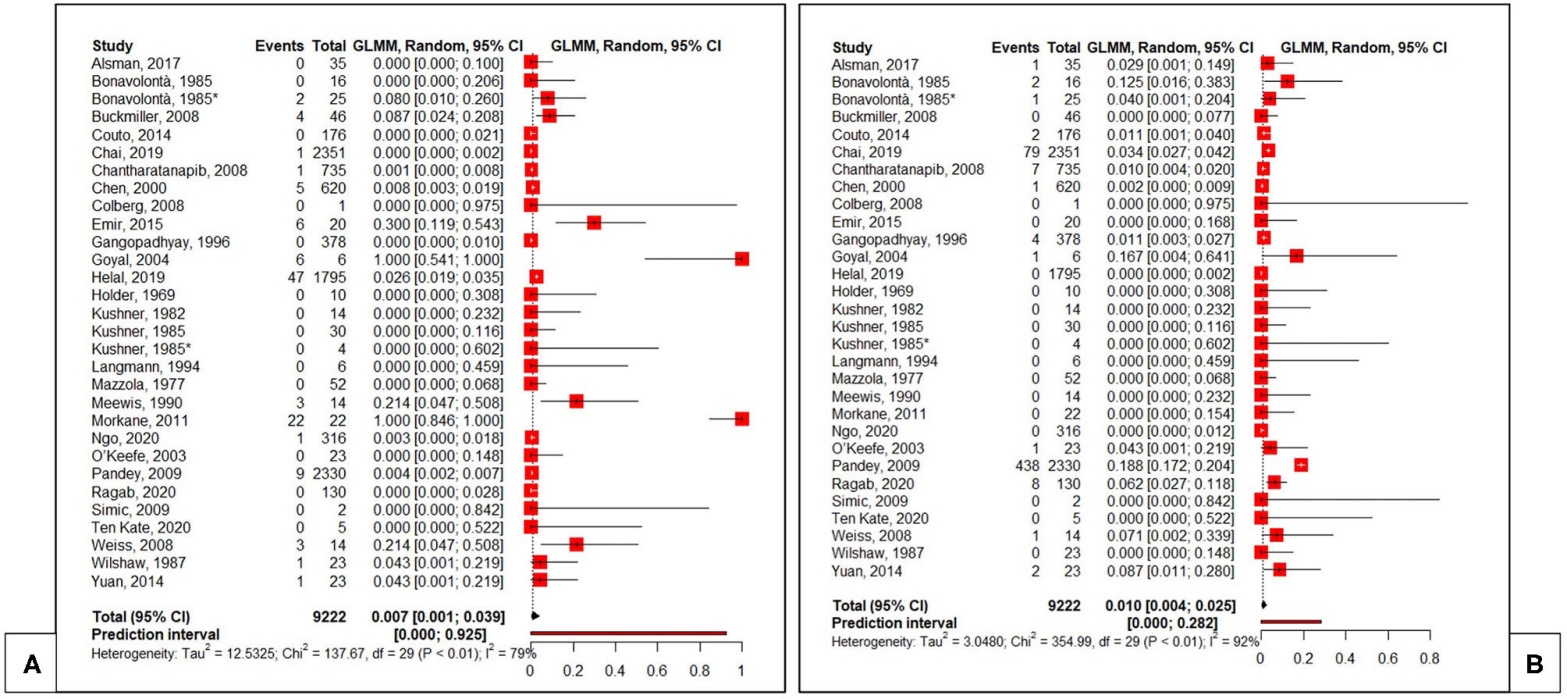
Pooled proportions for systemic **(A)** and local **(B)** complications (12, 34–60). Total, total number of injections. *Patients receiving ≥80 mg Triamcinolone Acetonide.

The pooled rates of local and systemic side effects were determined. A total of 730 side effects were seen, consisting of 139 systemic and 591 local effects. As indicated in Figure 3, the pooled local complication rate was estimated at 10% (95% CI 0.004–0.025), whereas the pooled systemic complication rate was estimated at 0.7% (95% CI 0.001–0.039). Further complication analysis, comparing the high and low dose groups, was not performed as the data retrieved in this systematic review did not allow further statistical analysis.

#### Systemic Complications

**Figure 4:**
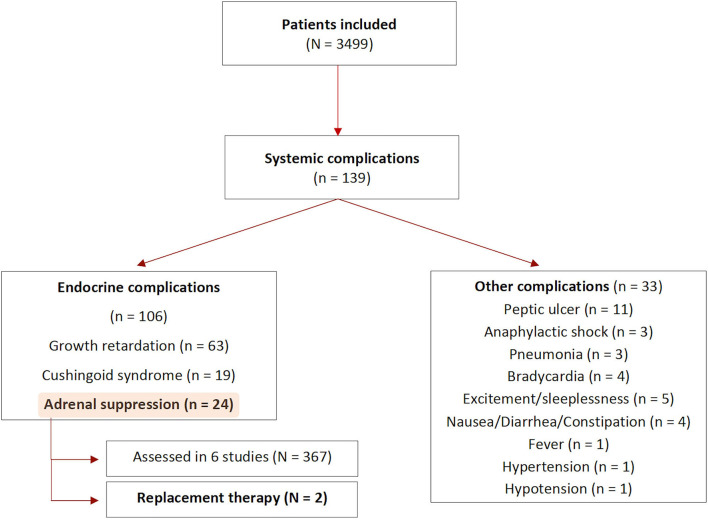
Summary of the systemic complications. N, number of patients; n, number of complications.

Eighteen studies reported systemic complications, of which sixteen referred to endocrinological side effects (see Figure 4) (34, 36–40, 43, 45, 46, 52, 53, 55, 58–61, 81, 82, 84, 86). A total of 139 systemic complications were described in 3,499 patients at risk, with 106 being related to adrenal insufficiency, Cushingoid syndrome or growth retardation. Among patients experiencing systemic complications, the indications for treatment were mostly periorbital or cutaneous hemangiomas, with only one study using ISI for the management of esophageal strictures.

The assessment of complications varied between studies. Forty-four studies only provided general clinical information, five studies evaluated patients' growth curves and six studies performed blood test analysis before and after the injection to rule out the development of adrenal insufficiency.

Among the six studies assessing cortisol and ACTH levels (367 patients), four found adrenal suppression following ISI (351 patients), mainly associated to weight loss or growth retardation (43, 45, 46, 53, 59, 84). One study reported the development of Cushingoid syndrome in seven patients (46). Only two out of 351 patients received replacement therapy (being oral hydrocortisone). All patients underwent strict clinical and laboratory follow-up. In all cases, height and weight percentiles returned to pre-treatment levels after pituitary-adrenal axis recovery. The time needed for hypothalamic-pituitary axis (HPA) recovery after ISI ranged between 4 and 65 weeks in the included studies. One study assessed HPA functioning in only one patient, who was re-admitted 3 weeks after the ISI due to a bronchopneumonia. The blood levels of ACTH and cortisol resulted within the normal range, the patient died due to non ISI-related complications (59).

### Effectiveness of ISI

The mean age of the patients included in the effectiveness analysis was 13.72 months. Only one study specified the type of esophageal atresia, with five patients being affected by type C and one patient by type D (12).

The indication for dilatation plus ISI was dysphagia in three studies (12, 34, 89) and failure to thrive in one study (88). One study did not explicitly mention the reason for dilatation (47). All patients but one had a history of dilatations and/or refractory stricture. Mean stricture length, based on data from three studies, was < 1.5 cm (47, 88, 89). All patients received one or multiple injections of 40 mg of triamcinolone acetonide or less (range 8–40 mg).

The injections were performed within four quadrants at the level of the esophageal stricture in all studies. In two studies, more than one injection was scheduled independently from the patients' symptoms (12, 89), while in the remaining studies the number of injections was individualized. The mean number of injections per patient was 2.1. In all studies ISI were performed directly before an endoscopic dilatation, carried out either with balloon or bougie. There was a minimum interval of 1 week between one ISI and the following one (88).

Referring to side effects, only one patient (0.58%) experienced transient adrenal insufficiency after the steroid injection, yet no additional treatment was required (34). No local side effects were defined. The follow-up length was mentioned in two studies, respectively, being 22.5 and 36 months (88, 89).

Four studies stated the number of ISI required or planned and the number of dilatations needed for each patient before and after the first injection, as shown in Table 2 (12, 47, 88, 89). The mean and median number of dilatations needed before ISI were 5.2 and 3, respectively. After ISI the mean and median number of further dilatations required were, respectively, 1.13 and 0. Overall, a mean of 7.53 dilatations and 1.67 ISI per patient were performed.

**Table 2:** Summary of dilatations and ISI per patient.

**Patient**	**Article (*N* = patients)**	**Dilatations prior to ISI (N)**	**Dilatations ±ISI after first ISI (N)**	**Total ISI (*N*)**	Subsequent ISI scheduled after first ISI^*^(yes/no)
1	Holder et al. (47)	7	2	3	Unclear
2	(*N* = 3)	12	1	2	Unclear
3		0	2	3	No
4	Gandhi et al. (88)	2	0	1	NA
5	(*N* = 5)	2	5	4	No
6		4	0	1	NA
7		0	0	1	NA
8		0	0	1	NA
9	Ten Kate et al. (12)	7	5^**^	2	Yes
10	(*N* = 6)	19	0	1	NA
11		3	1	2	Yes
12		2	1	1	NA
13		1	1	1	NA
14		15	0	1	NA
15	Zein et al. (89) (*N* = 1)	4	1	2	Yes

* *ISI injection planned after first ISI as standard protocol, independent from possible symptoms;*

** *5 dilatations and multiple esophageal stents*.

One study was not included in the analysis due to the lack of data referring to the number of dilatations prior to ISI (34). This study does show a significant difference in the esophageal diameter between patients receiving dilatation plus ISI and those receiving dilatations only, with the former group achieving a bigger diameter after treatment. Nevertheless, this difference was not sustained after three injections.

## Discussion

### Summary of Main Results

This systematic review aimed to determine the safety of ISI in young children and their effectiveness in anastomotic strictures after EA repair. The studies included in this systematic review and meta-analysis widely varied in terms of study design, population, and outcome. Different therapeutic approaches were used, with no standardized protocols neither for different treatment indications nor within the same disease group. As a consequence, a wide range of dosages and mean number of injections have been reported.

The literature review shows that the reported side effects following ISI are mostly local and do not require additional treatment. Furthermore, the correlation between side effects and ISI was difficult to properly evaluate, as for some cases the adverse event could also be a possible evolution of the disease itself (e.g., ulceration for cutaneous hemangiomas).

The meta-analysis, performed to define the safety of ISI, demonstrated a trend toward a higher rate of local complications (10%) compared to systemic complications (0.7%). Regrettably, available data did not allow to determine whether complications were dose-dependent.

Referring to the systematic review on the ISI as adjuvant treatment for esophageal strictures, only limited data on the outcome was available. Among the included studies, differences were found in the timing of the first injection, with great variation in the number of dilatations prior to the intervention. Moreover, the steroid dosage and the number of further injections or dilatations widely varied. The mean number of esophageal dilatations prior to ISI was found to be remarkably higher than the mean number of dilatations needed after the first steroid injection (5.2 vs. 1.13).

### Potential Biases in the Review Process

The quality of the available evidence, both on safety and effectiveness of ISI in the pediatric population is poor. Intralesional steroid injections are not yet a routine clinical application and the incidence of the treated diseases is low. In order to incorporate a large group of patients for this systematic review, studies with wide variance in terms of methodology were included and no limitation on publication date was defined. Additionally, all studies describing original patient data were included with no restriction on the study type. As a consequence, the overall quality of the studies might have been negatively influenced.

Comparison between studies was hampered by the fact that the majority aimed to assess effectiveness of ISI and therefore did not evaluate safety as primary outcome. Subsequently, modalities for safety assessment varied greatly between the studies.

### Agreements and Disagreements With Other Studies or Reviews

#### Safety of ISI

The lack of standardized protocols for ISI plays a key role in their limited use in the pediatric population. Many research questions are still to be addressed, including the timing, dosage, and maximum number of injections.

Current therapeutic indications involving the use of intralesional steroids often require more than one injection to achieve clinical improvement. The existing literature on esophageal strictures describes a mean of 2.5 ± 1.1 injections per patient (90). In line with available evidence, in this systematic review patients received a mean of 2.9 injections, however with some of them reaching over 10 local steroids injections. Since there is no clear safety assessment for ISI, multiple injections raise concerns on the potential cumulative effect. HPA suppression, Cushingoid syndrome and growth retardation are the main clinical manifestations potentially resulting from steroid administration (91).

With the data obtained in this systematic review it was not possible to properly evaluate the correlation between the steroid dosage or the number of injections and the development of side effects. Existing literature on this topic provides conflicting results. Complicating furthermore, steroid dosage, type of steroid injected and number of injection sites have been proven to influence the time needed for recovery when systemic clinical symptoms occur (92, 93).

One study did not describe an association between steroid dosage and the development of adrenal suppression, but a longer recovery time when two or more injections were administered (53). On the other hand, Potter et al. found that the duration of adverse events seem to be dose-dependent (94).

Triamcinolone is a lipophilic steroid, biologically inactive until it is deacetonized and rendered hydrophilic. On this basis, a local long-lasting effect and a lower concentration of systemic dissemination can be hypothesized when compared to other steroids (91).

Additionally, studies based on salivary cortisol levels' evaluation suggest that TA exhibits an exponential clearance, resulting in transient and short-lasting complications even for higher dosages. Available evidence on older children and adults describe a time range of 1–4 weeks before symptom regression or HPA function normalization takes place (91, 92, 94). In the literature included in this systematic review, a wider range has been found (53).

Despite a longer time for HPA-recovery, only seven out of 367 patients tested for adrenal suppression experienced Cushingoid syndrome, while the majority developed growth retardation. One study reports a 100% rate of adrenal suppression but only 50% of the patients received replacement therapy (45). After adrenal recovery, no differences were found between patients receiving supplementary treatment and those being strictly monitored. All other studies in this review, assessing adrenal insufficiency, did not provide any replacement therapy. The adrenal function recovered in all patients during the follow-up period and no differences were found in weight and height percentiles compared to pre-treatment levels (43, 45, 46, 53, 84).

One study found ACTH and cortisol concentrations to be within normal ranges for all patients, showing no adrenal suppression (46). A difference seen in the ISI technique compared to other studies, measuring ACTH and cortisol levels, was the application of local pressure for 5 min after the injection, but the exact role and influence of this maneuver cannot be assessed.

#### Effectiveness of ISI for Anastomotic Esophageal Strictures

The mean age of the EA patients was remarkably higher in comparison with other therapeutic indications (13.72 vs. 6.9 months). This can be explained by the fact that ISI are widely used in diseases like hemangiomas and most of the times represent the first therapeutic option. Esophageal atresia patients, on the contrary, usually undergo several dilatations before an adjunct endoscopic treatment is considered, as ISI are being reserved for complex (refractory or >2 cm) or recurrent anastomotic strictures (4, 8).

Whether the combination of ISI and dilatations improve the outcome, compared to dilatations alone, is still not clear. Different parameters are used to define the effectiveness, such as the need for further dilatation, the number of further dilatations, the frequency of further dilatation, the re-stricture-free survival, the mean time required to solve the stricture and the length of the dysphagia-free period. These endpoints are mainly derived from studies on esophageal strictures in adult patients (95–97). Main benchmarks for anastomotic esophageal strictures in EA patients are the need for further dilatations after ISI, their number and their frequency. Kochhar et al. defined a periodic dilatation index (PDI), evaluating the number of dilatations over time and demonstrating a lower need for dilatations over the months following ISI (20). Also, a lower PDI score reflected an increased interval between further dilatations, independently from the number of dilatations prior to the steroid injection.

This systematic review shows a decrease in the number of dilatations after ISI compared to the number of dilatations prior to the first injection. Nevertheless, defining a causative relationship from available data is difficult, mainly due to the small sample size, study methodology and to multiple confounding factors. Studies involved in the analysis describe symptom improvement after ISI and the reaching of a long-lasting dysphagia-free period after one or more injections. This finding is in line with other studies on esophageal strictures with different etiologies (21), but no score for dysphagia classification has been used in the included studies (98).

Apart from the small sample size, differences in inclusion criteria and study heterogeneity are primary limiting factors. Wide variation in the use of ISI can be found, especially referring to dosage, number of dilatations prior to the injections and number of injections. Some authors scheduled more than one injection independently from the patient symptoms, whereas others scheduled further injections only if additional dilatations were necessary while others preferred a single-time injection. Existing literature on this aspect is ambiguous, as some studies report high effectiveness even for low steroid dosages and single injection (20, 95, 99), while others did not see any symptom improvement, even with higher dosages (19, 100). Overall, the average dosage of steroids, both for single and recurrent injections, was found to be much lower for esophageal strictures compared to other indications (max. 40 mg vs. max. 400 mg).

Another possible confounding factor in the effectiveness assessment is the esophageal dilatation technique, since the use of either balloon or bougie might influence the outcome after dilatation (101). Due to the limited sample size, no comparative meta-analysis could be performed in the present review. For the same reason no correlation with antacid treatment, known to influence the stricture development or the recurrence rate, has been evaluated (101).

Lastly, it is worth considering that the number of dilatations for anastomotic esophageal strictures generally follow an hyperbolic course. Patients with refractory strictures will likely need further, recurrent dilatations to keep symptoms under control (3). This systematic review highlights a change of this trend in patients receiving ISI, which might suggest the effectiveness of the technique.

Consensus was found on the technique used for injection, with all studies dividing the amount of steroid into four quadrants at the level of the stricture, as proposed by Ramage et al. (18).

For esophageal strictures, no patient received more than 3 injections, in accordance with the safety cut-off described in the literature for both systemic and local effects (101).

### Implication for Practice

One of the primary aims of this meta-analysis and systematic review was to assess the safety of ISI. Due to the heterogeneity of the included studies, defining the real impact of this treatment on young children's health is difficult. Even though the retrieved data belong to studies with high variance in dosages, clinical practices, and time-ranges, the low occurrence of side effects is encouraging and does not compellingly show clinical complications. Based on the current data analysis, administration of less than three injections with a total steroid dosage under 80 mg is suggested to be safe for all indications.

Results from this systematic review show a reduction in the need for dilatations and therefore address ISI as effective (adjuvant) treatment for esophageal strictures.

### Implication for Research

This systematic review highlights the lack of sufficient data on the safety and effectiveness of ISI in young children. The potential benefit of this treatment for esophageal stenosis plays a key role in reducing the burden of disease. Well-designed prospective studies aiming to specifically assess the safety and the effectiveness of ISI in EA are needed in order to implement this technique in the daily clinical practice.

## Conclusion

Based on the present systematic review and meta-analysis, including over 3,000 patients, we can assume that ISI are safe in young children under the age of 2 years and effective in the treatment of anastomotic esophageal strictures. Nevertheless, healthcare providers need to be aware of the possible adrenal suppression, although based on current data most side effects are local and self-limiting. Close clinical follow-up with at least growth curve evaluation is warranted in patients undergoing this therapeutic intervention. Caregivers should always be informed about possible signs of acute adrenal dysfunction.

In anastomotic strictures after EA repair, intralesional steroid injections seem to reduce the number of esophageal dilatations needed and their frequency, without the occurrence of local or systemic side effects. Their systematic use in anastomotic esophageal strictures has great potential to reduce the burden of disease. Prospective, comparative studies are needed before ISI can be defined as a safe and effective technique.

## Data Availability Statement

The original contributions presented in the study are included in the article/Supplementary Material, further inquiries can be directed to the corresponding author.

## Author Contributions

AH and RP provided study conception and design, data collection, statistical analysis, and writing—first draft and revised versions. FH provided statistical analysis and writing—editing and revision. JV provided study conception and design and writing—first draft and revised versions. All authors read and approved the final version of the article.

## Conflict of Interest

The authors declare that the research was conducted in the absence of any commercial or financial relationships that could be construed as a potential conflict of interest.

## Publisher's Note

All claims expressed in this article are solely those of the authors and do not necessarily represent those of their affiliated organizations, or those of the publisher, the editors and the reviewers. Any product that may be evaluated in this article, or claim that may be made by its manufacturer, is not guaranteed or endorsed by the publisher.
