# Supplementary material for: The Safety of Intralesional Steroid Injections in Young Children and Their Effectiveness in Anastomotic Esophageal Strictures—A Meta-Analysis and Systematic Review
Source: Front Pediatr. 2022 Jan 28;9:825030. doi: 10.3389/fped.2021.825030 (PMC8837747; doi:10.3389/fped.2021.825030)
Supplement: Supplementary file 1 [file Data_Sheet_1.docx]

Supplementary Material

# Supplementary Data

- 1. **Search Strategy**  used on 16 Augustus 2021

**Embase.com**

('steroid'/exp OR 'steroid therapy'/exp OR 'dexamethasone'/de OR 'methylprednisolone'/de OR 'triamcinolone acetonide'/de OR (steroid* OR corticosteroid* OR glucocorticoid* OR Dexamethasone* OR Methylprednisolone* OR Triamcinolone-acetonide*):ab,ti,kw) **AND** ('esophagus atresia'/de OR 'intralesional drug administration'/de OR 'local drug administration'/exp OR 'parenteral drug administration'/de OR 'central nervous system drug administration'/exp OR 'interstitial drug administration'/exp OR 'intraabdominal drug administration'/exp OR 'intraarticular drug administration'/exp OR 'intrabiliary drug administration'/exp OR 'intracardiac drug administration'/exp OR 'intracartilaginous drug administration'/exp OR 'intracaudal drug administration'/exp OR 'intracavernous drug administration'/exp OR 'intracavitary drug administration'/exp OR 'intracochlear drug administration'/exp OR 'intradermal drug administration'/exp OR 'intraductal drug administration'/exp OR 'intrahepatic drug administration'/exp OR 'intralesional drug administration'/exp OR 'intraluminal drug administration'/exp OR 'intralymphatic drug administration'/exp OR 'intramammary drug administration'/exp OR 'intramural drug administration'/exp OR 'intramuscular drug administration'/exp OR 'intraneural drug administration'/exp OR 'intraocular drug administration'/exp OR 'intraosseous drug administration'/exp OR 'intraovarian drug administration'/exp OR 'intrapancreatic drug administration'/exp OR 'intraperitoneal drug administration'/exp OR 'intraplantar drug administration'/exp OR 'intraprostatic drug administration'/exp OR 'intrarenal drug administration'/exp OR 'intrasplenic drug administration'/exp OR 'intratendinous drug administration'/exp OR 'intratesticular drug administration'/exp OR 'intrathoracic drug administration'/exp OR 'intratumoral drug administration'/exp OR 'intratympanic drug administration'/exp OR 'intraurethral drug administration'/exp OR 'intrauterine drug administration'/exp OR 'intravesical drug administration'/exp OR 'periarticular drug administration'/exp OR 'perineural drug administration'/exp OR 'periocular drug administration'/exp OR 'periodontal drug administration'/exp OR 'peritumoral drug administration'/exp OR 'regional perfusion'/exp OR 'retrobulbar drug administration'/exp OR 'soft tissue drug administration'/exp OR 'subcutaneous drug administration'/exp OR 'submucosal drug administration'/exp OR 'subplantar drug administration'/exp OR 'transtympanic drug administration'/exp OR 'ureteral drug administration'/exp OR (((esophag* OR oesophag*) NEAR/3 (atres* OR atret*)) OR intralesional OR ((local OR locally OR parenteral* OR subcutan* OR regional* OR central-nervous-system OR interstitial OR intraabdominal OR intraarticular OR intrabiliary OR intracardiac OR intracartilaginous OR intracaudal OR intracavernous OR intracavitary OR intracochlear OR intradermal OR intraductal OR intrahepatic OR intraluminal OR intralymphatic OR intramammary OR intramural OR intramuscular OR intraneural OR intraocular OR intraosseous OR intraovarian OR intrapancreatic OR intraperitoneal OR intraplantar OR intraprostat* OR intrarenal OR intrasplenic OR intratendinous OR intratesticular OR intrathoracic OR intratumoral OR intratympanic OR intraurethral OR intrauterine OR intravesical OR periarticular OR perineural OR periocular OR periodontal OR peritumoral OR regional-perfusion OR retrobulbar OR soft-tissue OR submucosal OR subplantar OR transtympanic OR ureteral OR intra-abdominal OR intra-articular OR intra-biliary OR intra-cardiac OR intra-cartilaginous OR intra-caudal OR intra-cavernous OR intra-cavitary OR intra-cochlear OR intra-dermal OR intra-ductal OR intra-hepatic OR intra-lesional OR intra-luminal OR intra-lymphatic OR intra-mammary OR intra-mural OR intra-muscular OR intra-neural OR intra-ocular OR intra-osseous OR intra-ovarian OR intra-pancreatic OR intra-peritoneal OR intra-plantar OR intra-prostat* OR intra-renal OR intra-splenic OR intra-tendinous OR intra-testicular OR intra-thoracic OR intra-tumoral OR intra-tympanic OR intra-urethral OR intra-uterine OR intra-vesical OR intracerebral OR intracranial OR intradural OR intrameningeal OR intraspinal OR intraaccumbal OR intralocus-coeruleus OR intraventral-tegmental OR intracerebroventricular OR intracortical OR intrahippocampal OR intrahypothalamic OR intranigral OR intrastriatal OR brain OR intra-cerebral OR intra-cranial OR intra-dural OR intra-meningeal OR intra-spinal OR intra-accumbal OR intra-locus-coeruleus OR intra-ventral-tegmental OR intra-cerebroventricular OR intra-cortical OR intra-hippocampal OR intra-hypothalamic OR intra-nigral OR intra-striatal OR spinal OR epidural OR intracisternal OR intradiscal OR intralumbar OR intramedullary OR intrathecal OR intra-cisternal OR intra-discal OR intra-lumbar OR intra-medullary OR intra-thecal OR joint OR intrasynovial OR intra-synovial OR billary OR intraatrial OR intrapericardial OR intra-atrial OR intra-pericardial OR heart OR transcardial OR trans-cardial OR intracavernos OR intrapenile OR intra-cavernos OR intra-penile OR intracutan* OR lymph-node OR ophthalmic OR eye OR intracameral OR intracorneal OR intrastromal OR intravitreal OR subconjunctival OR subretinal OR subtenon OR intra-cameral OR intra-corneal OR intra-stromal OR intra-vitreal OR bone OR intratibial OR intraulnar OR intra-tibial OR intra-ulnar OR intrafootpad OR intrapaw OR intra-footpad OR intra-paw OR intratubular OR intra-tubular OR intratumoural OR intra-tumoural OR centrotumoral OR centro-tumoural OR urethral OR uterus OR uterine OR intracervical OR endocervical OR cervical OR bladder OR intrabursal OR intra-bursal OR peri-ocular OR intragingival OR intra-gingival OR isolation-perfusion) NEAR/3 (drug* OR administrat* OR inject* OR infusion* OR deliver* OR dose OR dosage* OR medication* OR therap* OR treatment* OR chemotherap*))):ab,ti,kw) **AND** (child/de OR boy/de OR girl/de OR infant/exp OR 'preschool child'/de OR toddler/de OR pediatrics/exp OR childhood/exp OR 'child welfare'/de OR 'child development'/de OR 'child growth'/de OR 'child health'/de OR 'child health care'/exp OR 'child care'/exp OR 'child death'/de OR 'pediatric ward'/de OR 'pediatric hospital'/de OR 'pediatric anesthesia'/de OR 'pediatric intensive care unit'/de OR 'neonatal intensive care unit'/de OR 'prematurity'/de OR (infan* OR newborn* OR (new NEXT/1 born*) OR baby OR babies OR neonat* OR prematur* OR pre-matur* OR child* OR kid OR kids OR toddler* OR boy* OR girl* OR kindergar* OR pediatric* OR paediatric* OR suckling* OR PICU OR NICU OR PICUs OR NICUs):ab,ti,kw) **AND** ('observational study'/exp OR 'cohort analysis'/exp OR 'longitudinal study'/exp OR 'retrospective study'/exp OR 'prospective study'/exp OR 'epidemiological data'/de OR 'case control study'/de OR 'cross-sectional study'/de OR 'correlational study'/de OR 'family study'/de OR 'major clinical study'/de OR 'multicenter study'/de OR 'comparative study'/de OR 'follow up'/de OR 'clinical study'/de OR 'clinical article'/de OR 'clinical trial'/exp OR 'randomization'/exp OR 'intervention study'/de OR 'open study'/de OR 'controlled study'/de OR 'review'/exp OR 'systematic review'/exp OR 'meta analysis'/de OR (((observation* OR epidemiolog* OR famil* OR comparativ* OR communit* OR interven*) NEAR/6 (stud* OR data OR research)) OR cohort* OR longitudinal* OR retrospectiv* OR prospectiv* OR (national* NEAR/3 (stud*)) OR ((case OR cases OR match*) NEAR/3 control*) OR (cross NEXT/1 section*) OR correlation* OR multicenter* OR multi-center* OR follow-up* OR followup* OR clinical* OR trial OR random* OR review* OR meta-analy* OR (mixed NEAR/3 method*)):ab,ti,kw) NOT ((animal/exp OR animal*:de OR nonhuman/de) NOT ('human'/exp)) AND [english]/lim NOT ([Conference Abstract]/lim)

**Medline**

(exp Steroids/ OR (steroid* OR corticosteroid* OR glucocorticoid* OR Dexamethasone* OR Methylprednisolone* OR Triamcinolone-acetonide*).ab,ti,kf.) AND (Esophageal Atresia/ OR Infusions, Intralesional/ OR Infusions, Parenteral/ OR Infusions, Spinal/ OR exp Injections, Spinal/ OR Infusions, Intraventricular/ OR exp Infusions, Subcutaneous/ OR exp Injections, Subcutaneous/ OR Injection, Intratympanic/ OR exp Injections, Intra-Articular/ OR Injections, Intralesional/ OR Injections, Intralymphatic/ OR Injections, Intramuscular/ OR Injections, Intraperitoneal/ OR Injections, Intraventricular/ OR Administration, Ophthalmic/ OR Infusions, Intraosseous/ OR Administration, Intravesical/ OR Chemotherapy, Cancer, Regional Perfusion/ OR (((esophag* OR oesophag*) ADJ3 (atres* OR atret*)) OR intralesional OR ((local OR locally OR parenteral* OR subcutan* OR regional* OR central-nervous-system OR interstitial OR intraabdominal OR intraarticular OR intrabiliary OR intracardiac OR intracartilaginous OR intracaudal OR intracavernous OR intracavitary OR intracochlear OR intradermal OR intraductal OR intrahepatic OR intraluminal OR intralymphatic OR intramammary OR intramural OR intramuscular OR intraneural OR intraocular OR intraosseous OR intraovarian OR intrapancreatic OR intraperitoneal OR intraplantar OR intraprostat* OR intrarenal OR intrasplenic OR intratendinous OR intratesticular OR intrathoracic OR intratumoral OR intratympanic OR intraurethral OR intrauterine OR intravesical OR periarticular OR perineural OR periocular OR periodontal OR peritumoral OR regional-perfusion OR retrobulbar OR soft-tissue OR submucosal OR subplantar OR transtympanic OR ureteral OR intra-abdominal OR intra-articular OR intra-biliary OR intra-cardiac OR intra-cartilaginous OR intra-caudal OR intra-cavernous OR intra-cavitary OR intra-cochlear OR intra-dermal OR intra-ductal OR intra-hepatic OR intra-lesional OR intra-luminal OR intra-lymphatic OR intra-mammary OR intra-mural OR intra-muscular OR intra-neural OR intra-ocular OR intra-osseous OR intra-ovarian OR intra-pancreatic OR intra-peritoneal OR intra-plantar OR intra-prostat* OR intra-renal OR intra-splenic OR intra-tendinous OR intra-testicular OR intra-thoracic OR intra-tumoral OR intra-tympanic OR intra-urethral OR intra-uterine OR intra-vesical OR intracerebral OR intracranial OR intradural OR intrameningeal OR intraspinal OR intraaccumbal OR intralocus-coeruleus OR intraventral-tegmental OR intracerebroventricular OR intracortical OR intrahippocampal OR intrahypothalamic OR intranigral OR intrastriatal OR brain OR intra-cerebral OR intra-cranial OR intra-dural OR intra-meningeal OR intra-spinal OR intra-accumbal OR intra-locus-coeruleus OR intra-ventral-tegmental OR intra-cerebroventricular OR intra-cortical OR intra-hippocampal OR intra-hypothalamic OR intra-nigral OR intra-striatal OR spinal OR epidural OR intracisternal OR intradiscal OR intralumbar OR intramedullary OR intrathecal OR intra-cisternal OR intra-discal OR intra-lumbar OR intra-medullary OR intra-thecal OR joint OR intrasynovial OR intra-synovial OR billary OR intraatrial OR intrapericardial OR intra-atrial OR intra-pericardial OR heart OR transcardial OR trans-cardial OR intracavernos OR intrapenile OR intra-cavernos OR intra-penile OR intracutan* OR lymph-node OR ophthalmic OR eye OR intracameral OR intracorneal OR intrastromal OR intravitreal OR subconjunctival OR subretinal OR subtenon OR intra-cameral OR intra-corneal OR intra-stromal OR intra-vitreal OR bone OR intratibial OR intraulnar OR intra-tibial OR intra-ulnar OR intrafootpad OR intrapaw OR intra-footpad OR intra-paw OR intratubular OR intra-tubular OR intratumoural OR intra-tumoural OR centrotumoral OR centro-tumoural OR urethral OR uterus OR uterine OR intracervical OR endocervical OR cervical OR bladder OR intrabursal OR intra-bursal OR peri-ocular OR intragingival OR intra-gingival OR isolation-perfusion) ADJ3 (drug* OR administrat* OR inject* OR infusion* OR deliver* OR dose OR dosage* OR medication* OR therap* OR treatment* OR chemotherap*))).ab,ti,kf.) AND (exp Infant/ OR exp "Child Behavior"/ OR exp "Parent Child Relations"/ OR exp "Pediatrics"/ OR "Child Nutrition Sciences"/ OR "Infant nutritional physiological phenomena"/ OR exp "Child Welfare"/ OR "Child Development"/ OR exp "Child Health Services"/ OR exp "Child Care"/ OR "Child Rearing"/ OR exp "Child development Disorders, Pervasive"/ OR "Child Psychiatry"/ OR "Child Psychology"/ OR "Hospitals, Pediatric"/ OR exp "Intensive Care Units, Pediatric"/ OR (infan* OR newborn* OR (new ADJ born*) OR baby OR babies OR neonat* OR prematur* OR pre-matur* OR child* OR kid OR kids OR toddler* OR boy* OR girl* OR kindergar* OR pediatric* OR paediatric* OR preschool* OR suckling* OR PICU OR NICU OR PICUs OR NICUs).ab,ti,kf.) AND ("Observational Study"/ OR exp "Cohort Studies"/ OR "Health Surveys"/ OR exp "Epidemiologic Studies"/ OR "Case-Control Studies"/ OR "Cross-Sectional Studies"/ OR "Multicenter Study"/ OR "Comparative Study"/ OR "Clinical Study"/ OR "Clinical Trial"/ OR "Random Allocation"/ OR "Review"/ OR "Meta-Analysis"/ OR (((observation* OR epidemiolog* OR famil* OR comparativ* OR communit*) ADJ6 (stud* OR data OR research)) OR cohort* OR longitudinal* OR retrospectiv* OR prospectiv* OR population* OR (national* ADJ3 (stud* OR survey)) OR (health* ADJ3 survey*) OR ((case OR cases OR match*) ADJ3 control*) OR (cross ADJ section*) OR correlation* OR multicenter* OR multi-center* OR follow-up* OR followup* OR clinical* OR trial OR random* OR review* OR meta-analy* OR (mixed ADJ3 method*)).ab,ti,kf.) NOT ((exp animal/) NOT (human/)) AND english.la. NOT (news OR congres* OR abstract* OR book* OR chapter* OR dissertation abstract*).pt.

**Web of Science**

TS=(((steroid* OR corticosteroid* OR glucocorticoid* OR Dexamethasone* OR Methylprednisolone* OR Triamcinolone-acetonide*)) AND ((((esophag* OR oesophag*) NEAR/2 (atres* OR atret*)) OR intralesional OR ((local OR locally OR parenteral* OR subcutan* OR regional* OR central-nervous-system OR interstitial OR intraabdominal OR intraarticular OR intrabiliary OR intracardiac OR intracartilaginous OR intracaudal OR intracavernous OR intracavitary OR intracochlear OR intradermal OR intraductal OR intrahepatic OR intraluminal OR intralymphatic OR intramammary OR intramural OR intramuscular OR intraneural OR intraocular OR intraosseous OR intraovarian OR intrapancreatic OR intraperitoneal OR intraplantar OR intraprostat* OR intrarenal OR intrasplenic OR intratendinous OR intratesticular OR intrathoracic OR intratumoral OR intratympanic OR intraurethral OR intrauterine OR intravesical OR periarticular OR perineural OR periocular OR periodontal OR peritumoral OR regional-perfusion OR retrobulbar OR soft-tissue OR submucosal OR subplantar OR transtympanic OR ureteral OR intra-abdominal OR intra-articular OR intra-biliary OR intra-cardiac OR intra-cartilaginous OR intra-caudal OR intra-cavernous OR intra-cavitary OR intra-cochlear OR intra-dermal OR intra-ductal OR intra-hepatic OR intra-lesional OR intra-luminal OR intra-lymphatic OR intra-mammary OR intra-mural OR intra-muscular OR intra-neural OR intra-ocular OR intra-osseous OR intra-ovarian OR intra-pancreatic OR intra-peritoneal OR intra-plantar OR intra-prostat* OR intra-renal OR intra-splenic OR intra-tendinous OR intra-testicular OR intra-thoracic OR intra-tumoral OR intra-tympanic OR intra-urethral OR intra-uterine OR intra-vesical OR intracerebral OR intracranial OR intradural OR intrameningeal OR intraspinal OR intraaccumbal OR intralocus-coeruleus OR intraventral-tegmental OR intracerebroventricular OR intracortical OR intrahippocampal OR intrahypothalamic OR intranigral OR intrastriatal OR brain OR intra-cerebral OR intra-cranial OR intra-dural OR intra-meningeal OR intra-spinal OR intra-accumbal OR intra-locus-coeruleus OR intra-ventral-tegmental OR intra-cerebroventricular OR intra-cortical OR intra-hippocampal OR intra-hypothalamic OR intra-nigral OR intra-striatal OR spinal OR epidural OR intracisternal OR intradiscal OR intralumbar OR intramedullary OR intrathecal OR intra-cisternal OR intra-discal OR intra-lumbar OR intra-medullary OR intra-thecal OR joint OR intrasynovial OR intra-synovial OR billary OR intraatrial OR intrapericardial OR intra-atrial OR intra-pericardial OR heart OR transcardial OR trans-cardial OR intracavernos OR intrapenile OR intra-cavernos OR intra-penile OR intracutan* OR lymph-node OR ophthalmic OR eye OR intracameral OR intracorneal OR intrastromal OR intravitreal OR subconjunctival OR subretinal OR subtenon OR intra-cameral OR intra-corneal OR intra-stromal OR intra-vitreal OR bone OR intratibial OR intraulnar OR intra-tibial OR intra-ulnar OR intrafootpad OR intrapaw OR intra-footpad OR intra-paw OR intratubular OR intra-tubular OR intratumoural OR intra-tumoural OR centrotumoral OR centro-tumoural OR urethral OR uterus OR uterine OR intracervical OR endocervical OR cervical OR bladder OR intrabursal OR intra-bursal OR peri-ocular OR intragingival OR intra-gingival OR isolation-perfusion) NEAR/2 (drug* OR administrat* OR inject* OR infusion* OR deliver* OR dose OR dosage* OR medication* OR therap* OR treatment* OR chemotherap*)))) AND ((infan* OR newborn* OR (new NEAR/1 born*) OR baby OR babies OR neonat* OR prematur* OR pre-matur* OR child* OR kid OR kids OR toddler* OR boy* OR girl* OR kindergar* OR pediatric* OR paediatric* OR suckling* OR PICU OR NICU OR PICUs OR NICUs)) AND ((((observation* OR epidemiolog* OR famil* OR comparativ* OR communit* OR interven*) NEAR/5 (stud* OR data OR research)) OR cohort* OR longitudinal* OR retrospectiv* OR prospectiv* OR (national* NEAR/2 (stud*)) OR ((case OR cases OR match*) NEAR/2 control*) OR (cross NEAR/1 section*) OR correlation* OR multicenter* OR multi-center* OR follow-up* OR followup* OR clinical* OR trial OR random* OR review* OR meta-analy* OR (mixed NEAR/2 method*))) NOT ((animal* OR rat OR rats OR mouse OR mice OR murine OR dog OR dogs OR canine OR cat OR cats OR feline OR rabbit OR cow OR cows OR bovine OR rodent* OR sheep OR ovine OR pig OR swine OR porcine OR veterinar* OR chick* OR zebrafish* OR baboon* OR nonhuman* OR primate* OR cattle* OR goose OR geese OR duck OR macaque* OR avian* OR bird* OR fish*) NOT (human* OR patient* OR women OR woman OR men OR man))) AND DT=(Article OR Review OR Letter OR Early Access) AND LA=(English)

**Cochrane**

((steroid* OR corticosteroid* OR glucocorticoid* OR Dexamethasone* OR Methylprednisolone* OR (Triamcinolone NEXT/1 acetonide*)):ab,ti,kw) **AND** ((((esophag* OR oesophag*) NEAR/3 (atres* OR atret*)) OR intralesional OR ((local OR locally OR parenteral* OR subcutan* OR regional* OR (central NEXT/1 nervous NEXT/1 system) OR interstitial OR intraabdominal OR intraarticular OR intrabiliary OR intracardiac OR intracartilaginous OR intracaudal OR intracavernous OR intracavitary OR intracochlear OR intradermal OR intraductal OR intrahepatic OR intraluminal OR intralymphatic OR intramammary OR intramural OR intramuscular OR intraneural OR intraocular OR intraosseous OR intraovarian OR intrapancreatic OR intraperitoneal OR intraplantar OR intraprostat* OR intrarenal OR intrasplenic OR intratendinous OR intratesticular OR intrathoracic OR intratumoral OR intratympanic OR intraurethral OR intrauterine OR intravesical OR periarticular OR perineural OR periocular OR periodontal OR peritumoral OR (regional NEXT/1 perfusion) OR retrobulbar OR (soft NEXT/1 tissue) OR submucosal OR subplantar OR transtympanic OR ureteral OR (intra NEXT/1 abdominal) OR (INTRA NEXT/1 articular) OR (INTRA NEXT/1 biliary) OR (INTRA NEXT/1 cardiac) OR (INTRA NEXT/1 cartilaginous) OR (INTRA NEXT/1 caudal) OR (INTRA NEXT/1 cavernous) OR (INTRA NEXT/1 cavitary) OR (INTRA NEXT/1 cochlear) OR (INTRA NEXT/1 dermal) OR (INTRA NEXT/1 ductal) OR (INTRA NEXT/1 hepatic) OR (INTRA NEXT/1 lesional) OR (INTRA NEXT/1 luminal) OR (INTRA NEXT/1 lymphatic) OR (INTRA NEXT/1 mammary) OR (INTRA NEXT/1 mural) OR (INTRA NEXT/1 muscular) OR (INTRA NEXT/1 neural) OR (INTRA NEXT/1 ocular) OR (INTRA NEXT/1 osseous) OR (INTRA NEXT/1 ovarian) OR (INTRA NEXT/1 pancreatic) OR (INTRA NEXT/1 peritoneal) OR (INTRA NEXT/1 plantar) OR (INTRA NEXT/1 prostat*) OR (INTRA NEXT/1 renal) OR (INTRA NEXT/1 splenic) OR (INTRA NEXT/1 tendinous) OR (INTRA NEXT/1 testicular) OR (INTRA NEXT/1 thoracic) OR (INTRA NEXT/1 tumoral) OR (INTRA NEXT/1 tympanic) OR (INTRA NEXT/1 urethral) OR (INTRA NEXT/1 uterine) OR (INTRA NEXT/1 vesical) OR (INTRA NEXT/1 cerebral) OR (INTRA NEXT/1 cranial) OR (INTRA NEXT/1 dural) OR (INTRA NEXT/1 meningeal) OR (INTRA NEXT/1 spinal) OR (INTRA NEXT/1 accumbal) OR (INTRA NEXT/1 cerebroventricular) OR (INTRA NEXT/1 cortical) OR (INTRA NEXT/1 hippocampal) OR (INTRA NEXT/1 hypothalamic) OR (INTRA NEXT/1 nigral) OR (INTRA NEXT/1 striatal) OR (INTRA NEXT/1 cisternal) OR (INTRA NEXT/1 discal) OR (INTRA NEXT/1 lumbar) OR (INTRA NEXT/1 medullary) OR (INTRA NEXT/1 thecal) OR (INTRA NEXT/1 synovial) OR (INTRA NEXT/1 atrial) OR (INTRA NEXT/1 pericardial) OR (INTRA NEXT/1 cameral) OR (INTRA NEXT/1 corneal) OR (INTRA NEXT/1 stromal) OR (INTRA NEXT/1 vitreal) OR (INTRA NEXT/1 tibial) OR (INTRA NEXT/1 ulnar) OR (INTRA NEXT/1 footpad) OR (INTRA NEXT/1 paw) OR intracerebral OR intracranial OR intradural OR intrameningeal OR intraspinal OR intraaccumbal OR (intralocus NEXT/1 coeruleus) OR (intraventral NEXT/1 tegmental) OR intracerebroventricular OR intracortical OR intrahippocampal OR intrahypothalamic OR intranigral OR intrastriatal OR brain OR (intra NEXT/1 locus NEXT/1 coeruleus) OR (intra NEXT/1 ventral NEXT/1 tegmental) OR spinal OR epidural OR intracisternal OR intradiscal OR intralumbar OR intramedullary OR intrathecal OR joint OR intrasynovial OR billary OR intraatrial OR intrapericardial OR heart OR transcardial OR (trans NEXT/1 cardial) OR intracavernos OR intrapenile OR (intra NEXT/1 cavernos) OR (intra NEXT/1 penile) OR intracutan* OR (lymph NEXT/1 node) OR ophthalmic OR eye OR intracameral OR intracorneal OR intrastromal OR intravitreal OR subconjunctival OR subretinal OR subtenon OR bone OR intratibial OR intraulnar OR intrafootpad OR intrapaw OR intratubular OR (intra NEXT/1 tubular) OR intratumoural OR (intra NEXT/1 tumoural) OR centrotumoral OR (centro NEXT/1 tumoural) OR urethral OR uterus OR uterine OR intracervical OR endocervical OR cervical OR bladder OR intrabursal OR (intra NEXT/1 bursal) OR (peri NEXT/1 ocular) OR intragingival OR (intra NEXT/1 gingival) OR (isolation NEXT/1 perfusion)) NEAR/3 (drug* OR administrat* OR inject* OR infusion* OR deliver* OR dose OR dosage* OR medication* OR therap* OR treatment* OR chemotherap*))):ab,ti,kw) **AND** ((infan* OR newborn* OR (new NEXT/1 born*) OR baby OR babies OR neonat* OR prematur* OR child* OR kid OR kids OR toddler* OR boy* OR girl* OR kindergar* OR pediatric* OR paediatric* OR suckling* OR PICU OR NICU OR PICUs OR NICUs):ab,ti,kw)

**Google Scholar**

steroid|corticosteroid|glucocorticoid local|locally|parenteral infant|infants|baby|babies|toddler|toddlers|pediatric|paediatric cohort|"follow*up"|followup|clinical|trial|review

# Supplementary Table

**Table 3 Overview of the database search results***Science Citation Index Expanded (1975-present); Social Sciences Citation Index (1975-present); Arts & Humanities Citation Index (1975-present); Conference Proceedings Citation Index- Science (1990-present); Conference Proceedings Citation Index- Social Science & Humanities (1990-present); Emerging Sources Citation Index (2015-present) ** Manually deleted abstracts from trial registries

| **Database searched** | **via** | **Years of coverage** | **Records** | **Records after duplicates removed** |
| --- | --- | --- | --- | --- |
| Embase | Embase.com | 1971 - Present | 6690 | 6633 |
| Medline ALL | Ovid | 1946 - Present | 2709 | 826 |
| Web of Science Core Collection* | Web of Knowledge | 1975 - Present | 1633 | 388 |
| Cochrane Central Register of Controlled Trials** | Wiley | 1992 - Present | 259 | 239 |
| Other sources: Google Scholar | | | 200 | 167 |
| **Total** | | | **11491** | **8253** |

**Table 4** Reported safety outcomes of studies included in the meta-analysis.
(x-y) = min-max**; ± =** SD**;** Med = Median; IQR = interquartile range; TA = Triamcinolone Acetonide; BMZ = Betamethasone; MPRED = Methylprednisolone; DEX=Dexamethasone
*Steroid dosage converted to triamcinolone acetonide (mg); ** Standard co-medication received by at least the majority of the included patients;
***Median; ****Total dose received, note: not dose per injection.

| **Author, Year** | **Included patients (N)** | **Age at time of injection (months)** | **Type of steroid** | **Dosage at first injection (mg)** | **Converted dosage* (mg)** | **Number of injections (N)** | **Time interval between injections (weeks)** | **Co-medication**** | **Length of follow-up (months)** | **Local side effects (N)** | **Systemic side effects (N)** | **Need for treatment of side effect** | **ACTH and/or cortisol measured** |
| --- | --- | --- | --- | --- | --- | --- | --- | --- | --- | --- | --- | --- | --- |
| Abe, 1986 | 2 | 3.25 (2.5-4) | TA | 9; 18 | - | 6; 8 | 0.5 - 4 | - | 36; 48 | - | Growth delay (N=1) | No | No |
| Al-Mahdi, 2010 | 1 | 4.25 | TA + DEX | 40+8 | 82.67 | 1 | - | - | 12 | Iris hypopigmentation (N=1) | - | No | No |
| Alsman, 2017 | 33 | 4.9 ± 2.6 | TA | 4-40 | - | 1 (N=31)  2 (N=2) | - | Propranolol | 6 | Subconjunctival hemorrhage (N=1) | - | No | No |
| Bonavolontà (0-79mg) | 6 | 7 | TA (+BMZ) | 20 – 40+4 **** | 20 – 61.3 | 1 (N=4)  2 (N=1) 3 (N=1) | 1-8 | - | 6-22 | Whitish subcutaneous deposit (N=1) Subconjunctival hemorrhage (N=1) | - | No | No |
| Bonavolontà (≥80mg) | 9 | 6 |  | 80+1.5 – 240+24 **** | 88-368 | 2 (N=3) 3 (N=3)  5 (N=1)  6 (N=2) |  |  | 8-30 | Whitish subcutaneous deposit (N=1) | Cushing syndrome (N=2) | No | No |
| Buckmiller, 2008 | 21 | 4.2 (1.5-8) | TA + BMZ | 40+3 | 60 | 2.2 | 12 | - | 12.38 | - | Failure to thrive (N=1) | No | No |
| Couto, 2014 | 100 | 2.75 (0.75-7.5) | TA | 1.6 mg/kg (0.76-5.4 mg/kg) | - | 1 (N=49) 2 (N=33) 3 (N=13) 4 (N=3)  5 (N=2) | 1.8 (1-5) | - | 1-1.5 | Subcutaneous atrophy (N=2) | - | No | No |
| Chai, 2019 | 1039 | Med 131 (IQR 81-234) | TA | 1.5 mg/kg (0.7-1.9) | - | 1-12 | 4-6 | Lauromacrogol + propranolol | 24 | Hypopigmentation (N=18), skin atrophy (N=23), flush and rash around injection area (N=36), ulceration N=12 | Fever (N=2) | Yes | No |
| Chantharatanapib, 2008 | 129 | 1 - 24 | TA | 1-2mg/kg, max 60 | - | 5.7 | 4; 8; 12 | - | Min 14 | Ulceration (N=4), hypopigmentation (N=1), entropian N=1) | Peptic ulcer (N=1) | No | No |
| Chen, 2000 | 155 | 3.8 (2-24) | TA | 10-30 | - | 4 | 5 | - | 40 | Atrophy (N=5) | Cushingoid (N=2), anaphylactic shock (N=3) | Yes | No |
| Colberg, 2008 | 6 | 17.2 (6-24) | TA | 4 (N=1)  8 (N=3) 8 + 2^nd^ injection 8 (N=1) | - | 1 (N=5) 2 (N=1) | - | - | 11-52 | - | - | - | No |
| Droste, 1988 | 2 | 3.125 (1.75-4.5) | TA + BMZ | 20+3; 10+1,5 | 40;20 | 3 (3^rd^ with 40+6); 3 (3^rd^ with 20+3) | 4.5; 6.75 | - | 19; 18 | Crease (N=1) | - | No | No |
| Edmonson, 2010 | 1 | 12 | TA | 40 | - | 3 | - | - | 8 | - | - | - | No |
| Emir, 2015 | 6 | 7 (range 5-18) *** | TA | 2mg/kg | - | 2 (N=2) 3 (N=1)  4 (N=2)  5 (N=1) | 4 | Propranolol | 9 | - | Adrenal suppresion (N=6) | No | Yes |
| Folia, 2007 | 1 | 9 | TA | - | - | 1 | - | - | 15 | - | - | - | No |
| Fonseca, 2021 | 1 | 15 | TA | 120 | 120 | 1 | - | - | 12 | - | - | - | No |
| Gangopadhyay, 1996 | 105 | 7 (1-15) | TA | 1-2 mg/kg, max 60 | - | 3.6 | 1 | - | - | Atrophy N=1, ulceration N=1, injection N=2 | - | - | No |
| Gorst | 1 | 2.25 | TA | 10 | - | 3 | 1 | - | 15.75 | - | - | - | No |
| Goyal | 4 | 4 (3-6) | TA; TA + BMZ | 40; 30; 20+2; 20+2 | 40; 30; 33.33; 33.33 | 2; 2; 1; 1 | 1-9 | - | 2-9 | N=1 white area at injection site | Adrenal suppresion N=2, weight los N=2 | Yes | Yes |
| Helal, 2019 | 340 | 7.43 ± 6.04 (3-49) | TA | 1mg/kg + 0.2mg/kg | - | - | 5.28 ± 0.5 (range 1-7) | Bleomycin | 6 | Ulceration N=30, atrophy N=7, scarring N=10 | - | No | Yes |
| Hoeve, 1997 | 11 | 2.5 (0.25-5.25) | MPRED | 60 | 60 | 1 | - | - | 96 | - | - | - | No |
| Holder, 1969 | 4 | 11.7 (0.75-19) | TA | 40 | - | 3; 3; 3; 1 | 5-24 | - | - | - | - | - | No |
| Hoornweg, 2014 | 29 | Med 2.92 (IQR 2.53) | TA | 40 | - | 1 (N=8)  2 (N=13)  >2 (N=16) | 8 | Prednisolon, methylprednisolone | 2 | Bleeding (N=9), ulceration (N=7) |  | Yes | No |
| Janmohamed, 2011 | 34 | med  3.8 (10th percentile =2, 90th percentile =9) | TA + BMZ | 80+12 | 32-160 | 1 (N=29)  2 (N=5) | - | 2.5% phenylephrine and 0.5% tropicamide drops | 12 | periocular calcification (N=1) | - | Unclear | No |
| Kang | 1 | 2 | TA | 20 | - | 3 | 4 | - | 9 | - | - | - | No |
| Khamalrudin | 1 | 8 | TA | - | - | 1 | - | - | 64 | - | - | - | No |
| Kushner, 1982 | 9 | 3.64 (1.75-10) | TA + BMZ | 20+3 until 80+12 | 40 until 160 | 1.42 | 11.84 | - | - | - | - | - | No |
| Kushner, 1985 (79mg) | 21 | 4.86 (1.5-11) | TA + BMZ | 9+4 – 40+6 | 30.3 - 72 | 1 (N=4) 2 (N=18) | 11.92 | - | - | - | - | - | No |
| Kushner, 1985 (80mg) | 3 |  |  | 80 + 12 | 144 | 2 (N=2) 3 (N=1) |  | - | - |  |  |  |  |
| Langmann, 1994 | 4 | 1-3 | BMZ + BMZ | 4+5 or 8+10 | 60 or 120 | 1.5 | 6 | - | 21-26 | - | - | - | No |
| Mazzola, 1977 | 11 | 8.2 (3-24) | MPRED | 2mg/kg | - | 4.73 | 1 | - | 34.48 | - | - | No | No |
| Meeuwis, 1990 | 6 | 4 (2-6.5) | MPRED | 20-40 | 16-32 | 1 (N=3)  3 (N=2)  5 (N=1) | - | (Previous steroid IV administration N=2) | 39.6 | Transient acne and local fluffy hairgrowth (N=1) | Growth retardation (N=1), pneumonia (N=2) | Yes | No |
| Mohamed, 2020 | 26 | Med 1.9 (1.7-2.5) | BMZ | 10-40 mg/ml | - | - | 4 | - | 6 | Ulceration N=2 | - | No | No |
| Morkane, 2011 | 15 | 2.5-14 | TA / BMZ | 20-40 + 2 | 33.33-53.33 | 1 (N=8)  2 (N=7) | - | - | 8 | - | Adrenal suppresion N=13, fall in ACTH concentration, N=14 failure to gain weight | No | Yes |
| Nelson, 1984 | 2 | 13 (2-24) | TA + DEX | 80+16; 40+8 | 165.33;82.67 | 1 | - | - | 10; 6 | - | - | No | No |
| Neumann (79mg) | 2 | 2; 5.5 | TA + BMZ | 40+6 | 72 | 1 | - | - | 3.5; 6 | - | - | No | No |
| Neumann (80mg) | 1 | 3.75 |  | 60+9**** | 108 | 2 | 10 |  | 4.47 |  |  |  |  |
| Ngo | 158 | 11.75 (6-24) | TA | 20-40 | - | 2.9 | - | - | - | - | Hypotension and adrenalsufficiency (N=1) | No | No |
| Noe, 1981 | 2 | 8; 7 | TA | - | - | 3; 1 | 6 | - | 6; 15 | - | - | No | No |
| O’Keefe, 2003 | 14 | 6.64 (2-17) med 5 | MPRED + DEX | 20 +4 | 41.33 | 1 N=5, 2 N=9 | 4 | - | - | Ulceration (N=1) | - | Yes | No |
| Pandey, 2009 | 886 | 8.69 (1-49) | TA | 1-2mg/kg | - | 2.63 | 4 | - | 60 | Infection (N=103), atrophy (N=100), hypogigmentation (N = 91), ulceration (N=144) | Hypertension (N=1), growth retardation (N=4), cushingoid facies (N=4) | Unclear | No |
| Ragab, 2020 | 25 | 11 (6-24) | TA | 0.5 mg/kg | - | 5.2 ± 0.79 | 4-6 | - | 7 months (range 3 months to 12 months) | epithelial sloughing and ulceration (N=2), bleeding (N=1), atrophy (N=2), hypopigmentation (N=3) | - | No | No |
| Reyes, 1989 | 1 | 1.25 | TA | 4-12 | - | 5 | 2-3 | - | 18 | - | - | - | No |
| Sabry, 2020 | 15 | 3-9 | TA | Max. 2.5 mg/kg | - | - | - | - | 6 | Ulceration (N=2) | - | No | No |
| Say, 2011 | 1 | 16 | TA | 20 | - | 3 | 20 | Radiotherapy | 12 | Hypopigmentation and atrophy (N=1) | - | No | No |
| Sekioka, 2018 | 1 | 16 | TA | 8 |  | 10 | 3-4 | - | 4 | - | - | No | No |
| Shao, 2016 | 31 | 4-20 | BMZ | 0.05ml/cm2 up to 0.3ml | - | - | 4-6 | Oral propranolol | - | - | Heart rate decrease (N=4), excitement/sleeplessness (N=5), nausea/diarrhea/constipation (N=4), growth retardation (N=1) | No | No |
| Simic, 2009 | 5 | 5.6 (2.5-8.5) | TA + DEX | 40 +4 | 61.33 | 1 (N=2) 2 (N=3) | 1.17 | - | - | - | - | No | No |
| Sun | 35 | 3.3 (0.6-7) | BMZ | 0.5-1 ml | - | - | Min 4 | Oral propranolol | - | Atrophy N=3, ulceration N=1, | Cushing N=1 | No | No |
| Tasca, 2004 | 1 | 2 | TA | 20 | - | 1 | - | - | 6 | - | - | No | No |
| Ten Kate | 4 | Med 7.16 (2.07-14.07) | TA | 40 | - | 1 (N=3)  2 (N=1) | 2 | - | Minim 12 | - | - | No | No |
| Weiss, 1989 | 2 | 2 | TA + BMZ | 20+4; 20+3 | 46.67; 40 | 1 | - | - | 10; 6 | - | Adrenal suppression (N=2), growth retardation (N=1) | No | Yes |
| Weiss, 2008 | 13 | 4.7 ± 2.7 (2-10) | TA + BMZ | 20+2 | 33.33 | 1 (N=12)  2 (N=1) | - | - | 21 | Superficial eyelid necrosis (N=1) | Growth retardation (N=3) | No | No |
| Wilshaw, 1987 | 15 | 5-38 | TA or DEX or MPRED | 40 (TA), 4 (DM), 20 (MP) | 40; 21.33; 20 | 1.53 | 20 | - | 19 | - | - | - | No |
| Xu, 2018 | 39 | 3.98 (1.5-6) | BMZ | Max. 14 | Max. 93.33 | 1.5 | 4 | 0.5% topical timolol | 5 | Atrophy (N=1), ulceration (N=1), desquamation (N=1) | - | No | No |
| Yuan, 2014 | 16 | 3.1 (1-7) | BMZ | 3.5-7 | 23.33-46.67 | 1.44 | 8 | - | - | Atrophy (N=1), ulceration (N=1) | Cushing (N=1) | No | No |
| Yuan, 2015 | 57 | 3.9 (1-7) | BMZ | 3.5 - 14 | 23.33 -93.33 | 1.72 | 2 | - | 6-60 | Atrophy (N=3), ulceration (N=2) | Cushing-like manifestations (N=2) | No | No |
| Zhang, 2021 | 36 | 3.58 (1-6) | BMZ | - | - | 1.5 | - | Topic timolol | 9-24 | Atrophy (N=1) | - | No | No |
